# Cultural and urban city living expectations of knee motion in a Southeast Asian city: implications on total knee arthroplasty outcomes

**DOI:** 10.3389/fresc.2024.1446389

**Published:** 2025-01-07

**Authors:** Yong Yao Tan, Ke Xin Magneline Ang, Ching Yi Aliza Wong, Kinjal Vidyut Mehta, Sir Young James Loh

**Affiliations:** Department of Orthopaedic Surgery Changi General Hospital, Singapore, Singapore

**Keywords:** knee, arthroplasty, gait, kinetics, orthopaedic, surgery, rehabilitation

## Abstract

**Background:**

The prevalence of knee osteoarthritis in Southeast Asia has increased steadily over the years. When conservative management options fail, total knee arthroplasty (TKA) is a reliable surgical option. Despite over 90% post-operative satisfaction, the high volume of TKAs performed means that even a small percentage of dissatisfied patients holds significance. Dissatisfaction often arises from unmet expectations, which are influenced by cultural and urban factors specific to the region. Surgeons must counsel patients on likely post-operative activities, but limited data on knee range of motion (ROM) for daily tasks in Southeast Asia renders personalised guidance challenging.

**Aim:**

The primary aim of this study was to evaluate the minimum knee ROM required for daily activities (walking, stair climbing, squatting, kneeling, and sitting cross-legged) in a healthy Southeast Asian urban population. The secondary aim was to determine the association between anthropometric measurements and knee ROM required for the activities. This information is useful in managing patient expectations after TKA.

**Methods:**

An email describing the study was sent to hospital staff to recruit them as volunteers for the study. Enrolled participants were required to be between the ages of 21 and 65 years and capable of providing informed consent. Interested volunteers with a past medical history involving the lower limbs and spine were excluded. Those with a history of lower limb joint pain or back pain within the last 1 year were also excluded. The KINEXCS KIMIA device (KIMIA Recover System, 2022 edition v1.0) was used to measure the knee ROM. The overall characteristics of participants were summarised using descriptive statistics. Anthropometric measurements and their association with knee ROM during the various activities were analysed. Variables with *p*-values <0.3 in univariate analysis were included in the multivariable analysis.

**Results:**

Fifty participants (32 men, 18 women) were enrolled in the study. The average age of participants was 28.5 years (SD 5.57). The average height was 168 cm (SD 0.0870), and the average body mass index was 23.02 kg/m^2^ (SD 2.82). The average knee ROM requirements for various activities are as follows: walking on flat ground and stair climbing required less than 90°, squatting and kneeling required at least 127°, and sitting cross-legged required 106°. Lower limb length, leg length, and calf circumference were significantly correlated with the knee ROM required to perform the activities.

**Significance:**

The findings of this study, combined with an understanding of cultural expectations, might prove valuable for counselling and managing patients undergoing TKA in an urban Southeast Asian country.

## Introduction

In Southeast Asia, activities like squatting, kneeling, and sitting cross-legged are more commonly performed compared to other areas. Squatting is more prevalent for household chores, toileting, and various work-related activities, such as dishwashing (1). In this region, where the world's largest Muslim population resides, practitioners of Islam perform daily prayers that involve repetitive kneeling, sometimes up to 30 times a day (2). Sitting cross-legged for meals, meditation, and religious activities is also more widely practised among the Asian population (3). These activities, which require significant knee flexion, have been associated with a higher prevalence of knee osteoarthritis among individuals who regularly perform them (1).

Data from the Global Burden of Disease Study revealed that the prevalence of knee osteoarthritis in Southeast Asia has steadily increased over the years and is expected to remain a significant contributor to years lived with disability (4, 5). When conservative management fails, total knee arthroplasty (TKA) is one of the most predictable surgical solutions for patients with advanced knee osteoarthritis, with the primary goals of achieving pain relief and improving function. In Singapore, a multicultural and modern metropolis where the authors practice, TKA is a readily accessible treatment, with more than 4,000 cases reported annually (6). Local studies have shown that patient satisfaction following TKA exceeds 90% (7, 8).

With the high volume of TKAs performed, even a small proportion of dissatisfied patients becomes significant. While persistent pain is the most common source of dissatisfaction, limited knee flexion is another key factor (7, 8). The authors believe that dissatisfaction with knee range of motion (ROM) may stem from a mismatch between patient expectations and actual outcomes, an observation that has been well-reported in the literature (8, 9). In the unique context of an urban Southeast Asian city, this can be influenced by cultural factors and the presence of urban infrastructure like stairs.

To better align post-operative expectations after TKA, surgeons must counsel patients on the activities they can likely perform after the surgery. However, limited data on the knee ROM required for daily activities in Southeast Asia make individualised counselling challenging. Therefore, the primary aim of this study was to evaluate the minimum knee ROM required for daily activities in a healthy Southeast Asian urban population. This information is useful in managing patient expectations after TKA.

## Methods

### Study design

This was a case series conducted in a Singapore public hospital, with ethics approval obtained from the Institutional Review Board (IRB) of the authors’ affiliated institution.

### Participants

An email describing the study was sent to hospital staff to recruit them as volunteers for the study. Interested participants were required to fill a screening questionnaire to determine their eligibility for the study.

#### Inclusion criteria

All participants were required to be between the ages of 21 and 65 years old and capable of providing informed consent.

#### Exclusion criteria

The exclusion criteria included osteoarthritis of any lower limb joint, formally diagnosed or self-reported generalised ligamentous laxity, history of lower limb joint pain or back pain within the last year, history of any lower limb or spine fractures, and history of any procedure performed on the spine or lower limbs. Participants were not informed of the exclusion criteria prior to this to avoid bias in their reporting.

### Ethical considerations

Once an interested volunteer was deemed eligible based on the screening criteria, a study visit was scheduled. Informed consent was obtained from all participants after they were provided with an understanding of the aims, risks, and benefits of participation. Consent was also obtained for the publication of their anonymised data and images.

### Demographic data

Participants’ age and gender were collected as part of the demographic data collection.

### Anthropometric measurements

The anthropometric measurements recorded were height, weight, abdominal circumference (measured at the umbilicus), lower limb length (measured from anterior superior iliac spine to medial malleolus), leg length (head of the fibula to lateral malleolus), thigh circumference (10 cm superior to the proximal pole of the patella), and calf circumference (5 cm inferior to the distal pole of the patella). The height, abdominal circumference, lower limb length, leg length, thigh circumference, and calf circumference were recorded in centimetres. Weight was recorded in kilograms. The anthropometric measurements collected have been previously reported to influence knee ROM (10, 11).

### Primary outcome: knee ROM

**Figure 1:**
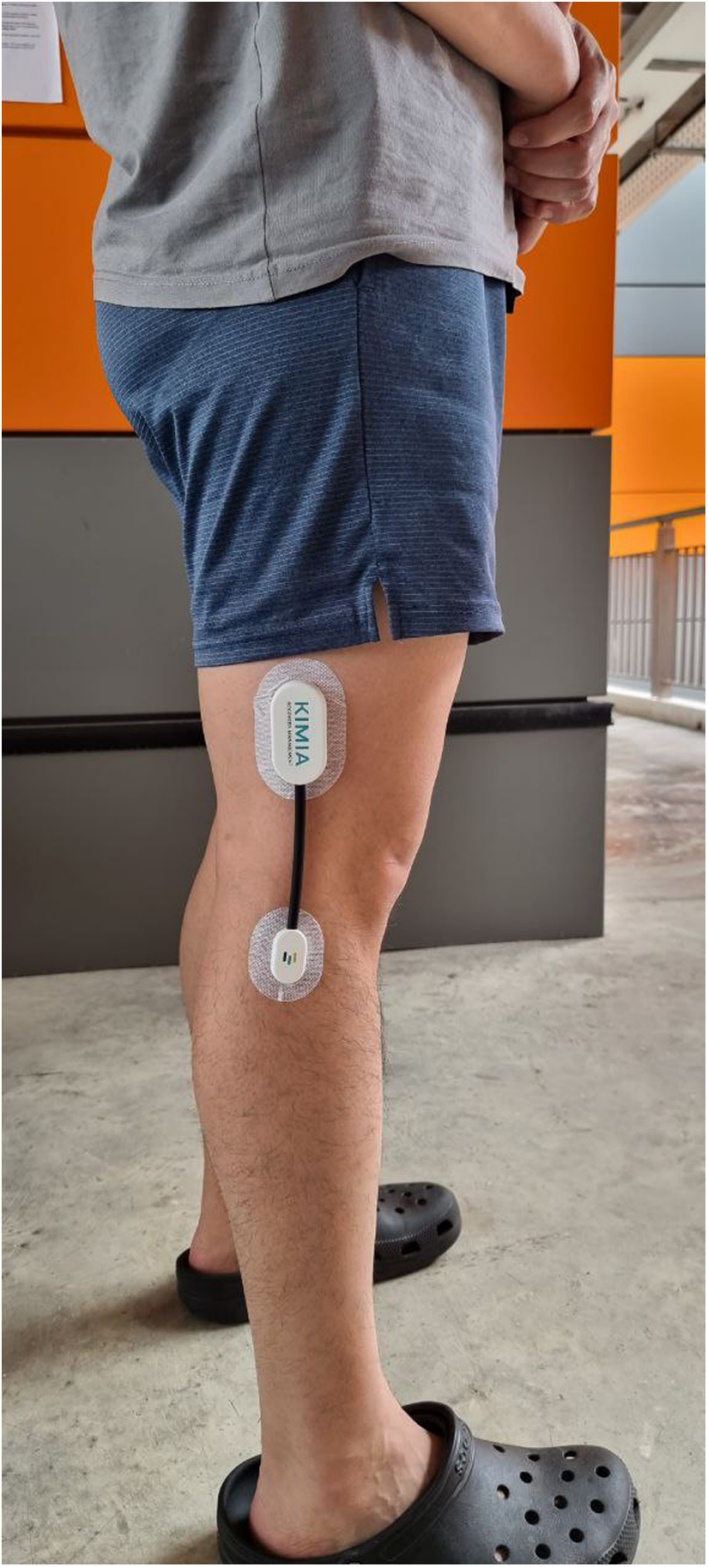
Kimia device attached to right lower limb.

The KINEXCS KIMIA device (KIMIA Recover System, 2022 edition v1.0) was used to measure the knee ROM. It was attached to the right knee according to the manufacturer's instructions (Figure 1). The device was positioned with the participant standing upright. The middle of the flexible tube was centred on the lateral femoral condyle. The larger end of the device was attached parallel to the thigh, while the smaller end was attached parallel to the shin. The device was attached to the skin using adhesive tape. Knee ROM was measured in degrees. The KINEXCS KIMIA is a device developed for kinematic measurement and analysis of joint movements. It consists of a sensor module that can be positioned across a patient's joint by attachment to disposable wearable adhesive patches. It measures joint angles using an integrated accelerometer and gyroscope sensor, with real-time readings displayed on a dedicated mobile application. Each activity was performed three times, and the minimum value was recorded.

### Activities evaluated

All participants wore either shorts or loose-fitting scrubs. Each activity was performed three times, and the minimum and maximum degrees of flexion were recorded.

#### Walk on flat ground

Participants were instructed to walk 5 m on flat ground. The degree of knee flexion was recorded at the peak of the swing phase when participants were walking on flat ground.

#### Ascend and descend stairs

For the stair climbing activity, one flight of stairs consisted of 13 steps, with each step measuring 15 cm in height and 28 cm in depth. The 15-cm step height was chosen because it is the shortest recommended dimension for standard precast staircases in Singapore, per the Building and Construction Authority (BCA) (12). The knee ROM recorded when participants were ascending or descending stairs represented the maximum flexion required throughout the activity.

#### Squatting and kneeling

**Figure 2:**
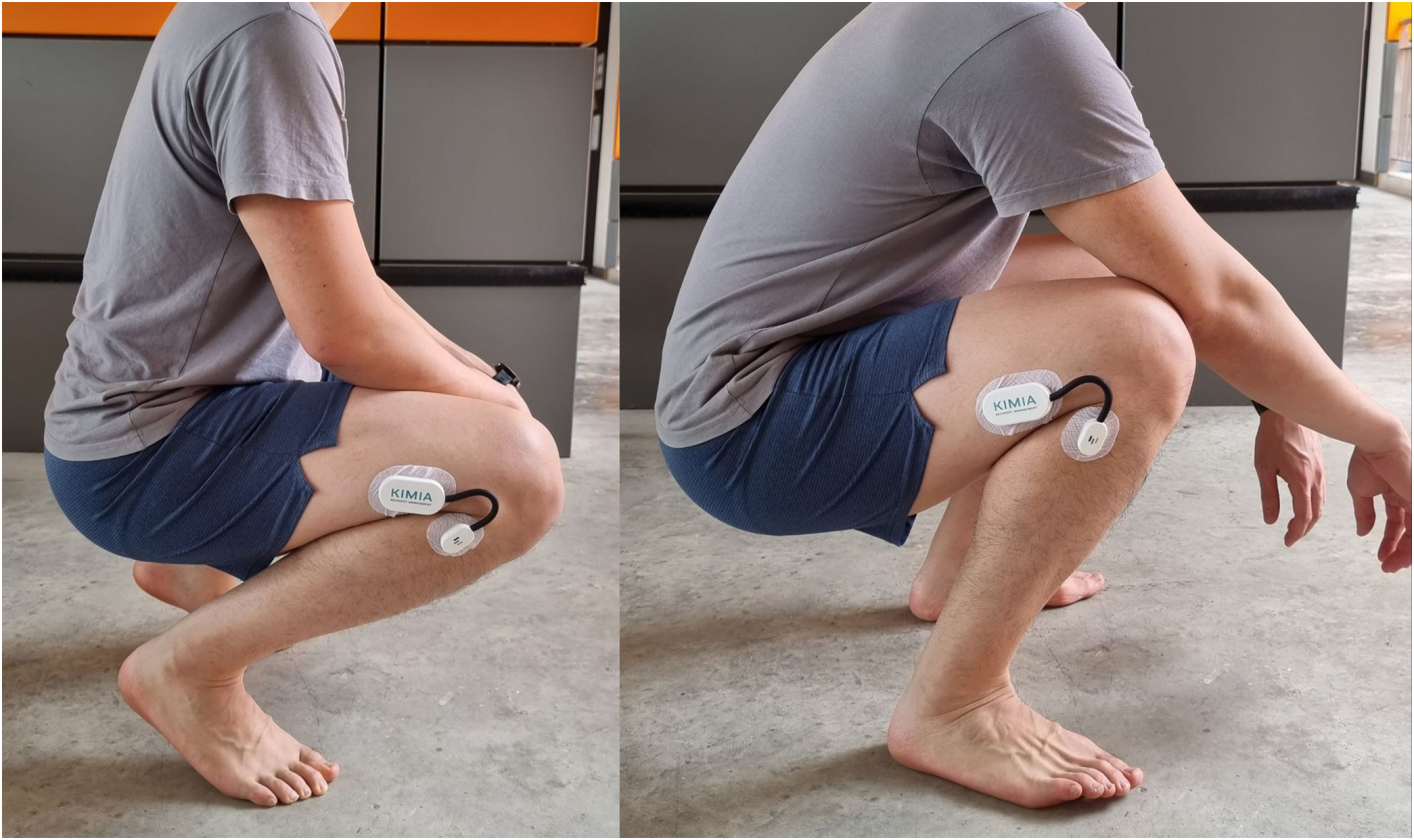
Participant squatting with heels raised and heels planted.

**Figure 3:**
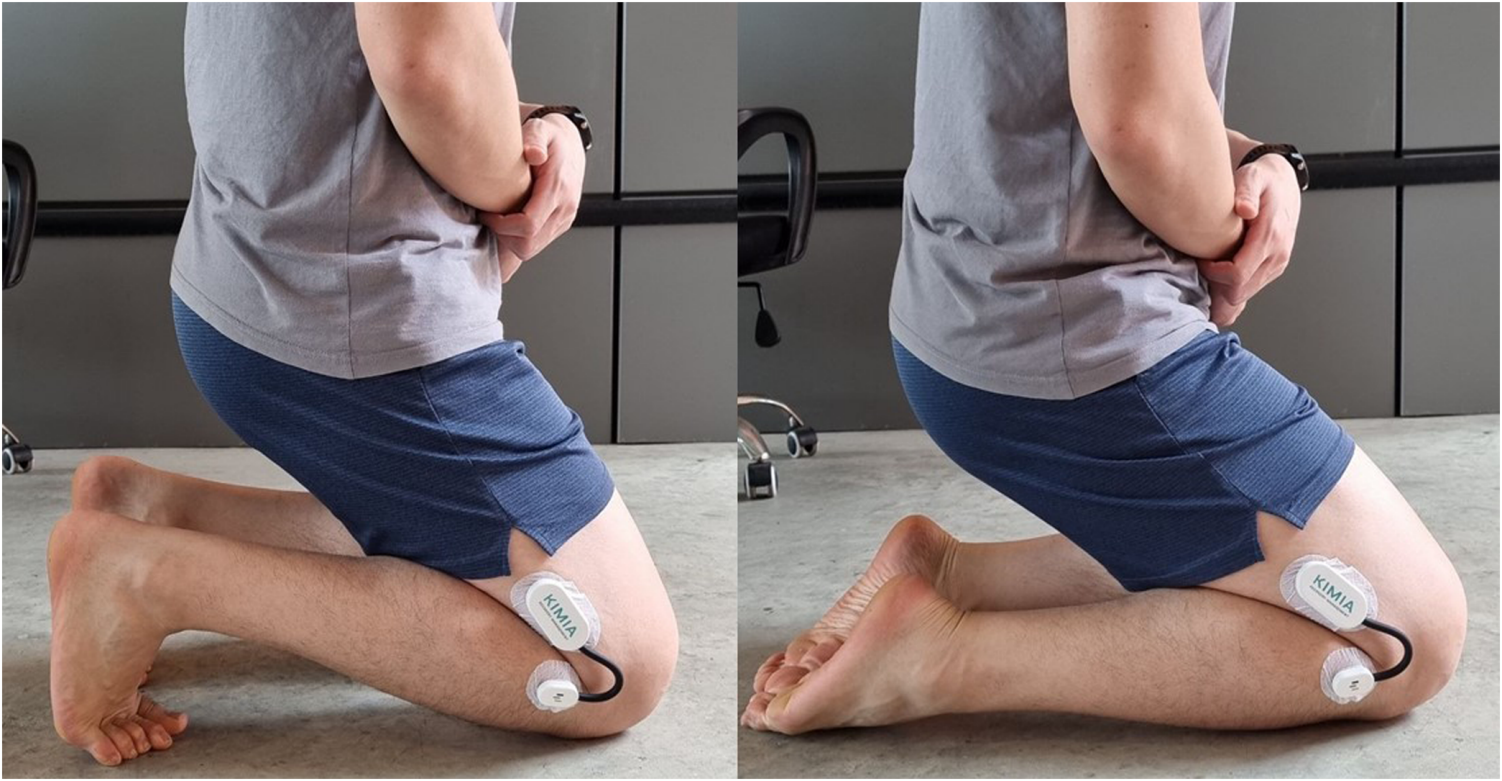
Participant kneeling with ankles dorsiflexed and plantarflexed.

For the squatting movement, participants were instructed to perform a squat with heels planted on the floor followed by a squat with heels raised (Figure 2). To evaluate the kneeling posture, participants were instructed to kneel in two positions: ankles dorsiflexed and ankles plantarflexed (Figure 3). During the squatting and kneeling activities, participants were instructed to bring their gluteus muscles as close to their heels as possible without causing discomfort in any of the lower limb joints. The readings were taken once participants were in the final position.

#### Sitting cross-legged

**Figure 4:**
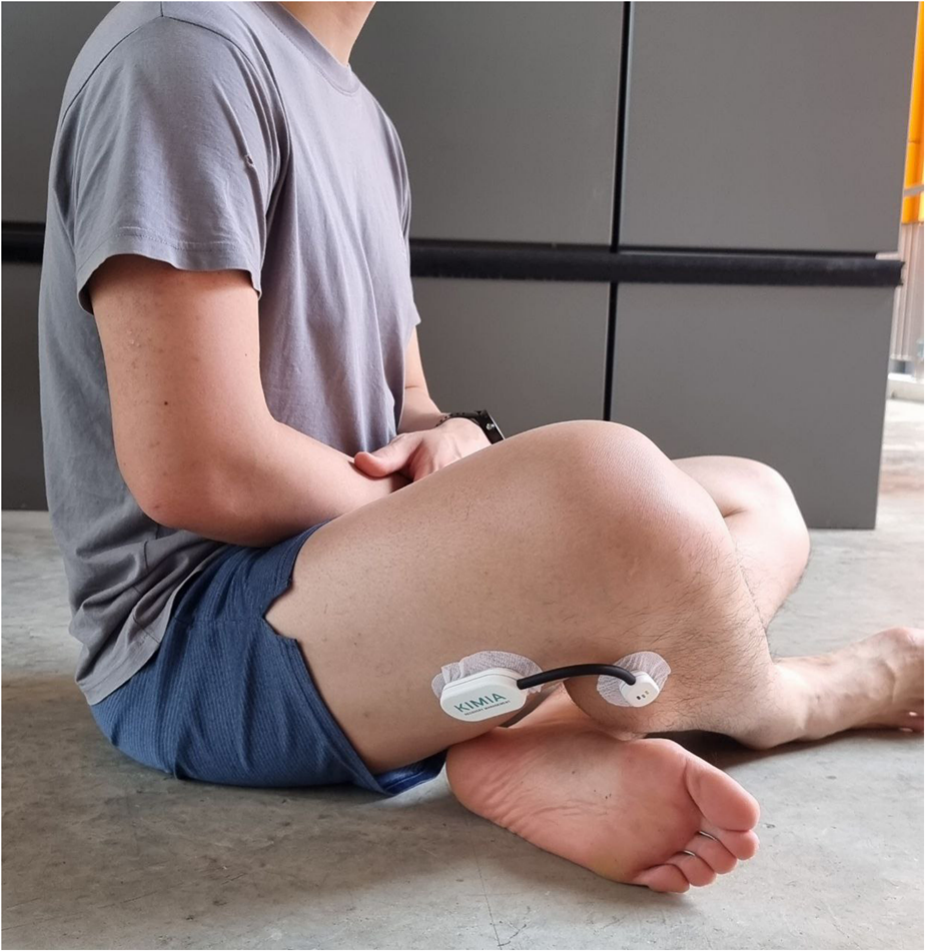
Participant sitting cross-legged.

Finally, the knee ROM was recorded when participants sat on the ground with their legs crossed (Figure 4). When sitting cross-legged, the medial aspect of the foot had to be in contact with the lateral aspect of the contralateral calf.

### Data analysis

The overall characteristics of participants were summarised using descriptive statistics, with continuous variables reported as mean ± standard deviation (SD). Anthropometric measurements and their association with knee ROM during the various activities were analysed using Pearson correlation or Spearman rank correlation. Variables with *p*-values <0.3 in the univariate analysis were selected for inclusion in a multivariable linear regression analysis. All statistical analyses were performed using Stata version 13 (StataCorp LP, College Station, TX), with statistical significance set at a *p*-value of <0.05.

## Results

A total of 50 participants were enrolled in the study, comprising 32 men and 18 women. The average age of participants was 28.5 years (SD 5.57). The average height was 168 cm (SD 0.0870), and the average body mass index (BMI) was 23.02 kg/m^2^ (SD 2.82). The anthropometric measurements are listed in Table 1.

**Table 1:** Biodata and anthropometric measurements.

	All, mean (SD)	Male, mean (SD)	Female, mean (SD)
Number of participants	50	32 (64%)	18 (36%)
Age (years)	28.5 (5.57)	26.9 (2.64)	31.3 (7.97)
Height (cm)	168 (0.0870)	173 (0.0576)	1.59 (0.0525)
Weight (kg)	65.5 (12.1)	71.4 (0.5)	54.9 (5.86)
BMI (kg/m^2^)	23.02 (2.82)	23.8 (2.74)	21.7 (2.48)
Abdominal circumference (cm)	82.4 (9.70)	86.5 (8.52)	75.0 (7.09)
Right lower limb length (cm)	91.4 (6.02)	93.9 (5.50)	87.1 (4.20)
Right leg length (cm)	39.3 (3.46)	39.9 (3.50)	38.1 (3.18)
Right thigh circumference (cm)	44.6 (3.54)	45.4 (3.20)	43.2 (3.77)
Right calf circumference (cm)	35.2 (3.11)	36.2 (3.16)	33.6 (2.25)

The knee range of motion described represents the functional segment of its full range required for the tested activity and may not involve terminal flexion or extension. The average knee ROM required for walking on flat ground was 55.7° (SD 8.76). The average knee ROM values required for ascending and descending stairs were 68.2° (SD 12.0) and 70.9° (SD 16.6), respectively. The average knee ROM values required for squatting with heels planted and heels raised were 127° (SD 14.1) and 131° (SD 14.0), respectively. The average knee ROM values required for kneeling with ankles dorsiflexed and plantarflexed were 133° (SD 15.0) and 130° (SD 14.4), respectively. Finally, the average knee ROM required for sitting cross-legged was 106° (SD 12.6).

### Association between knee ROM and anthropometric measurements

**Figure 5:**
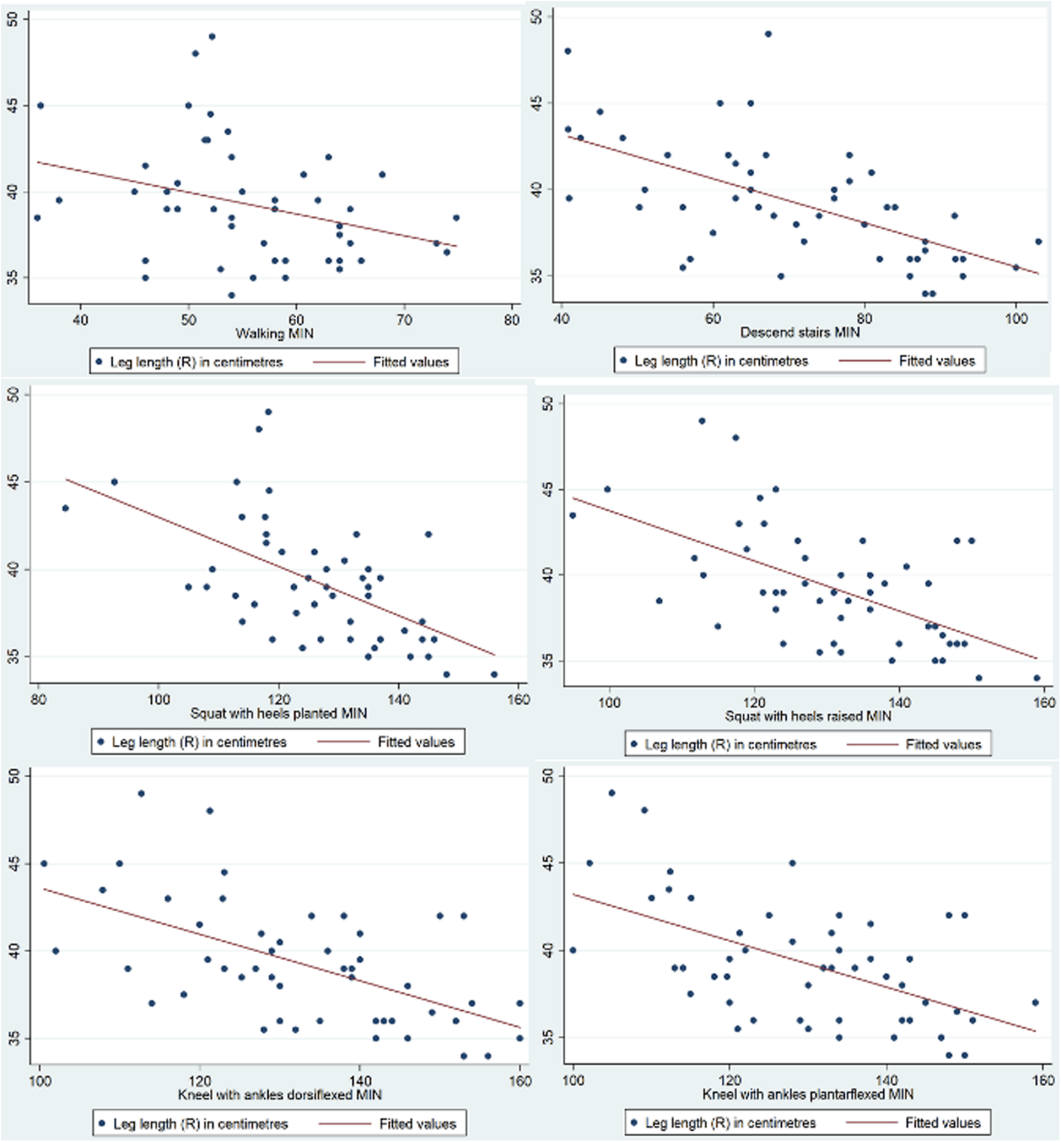
Correlation between leg length and knee range of movement during activities.

**Figure 6:**
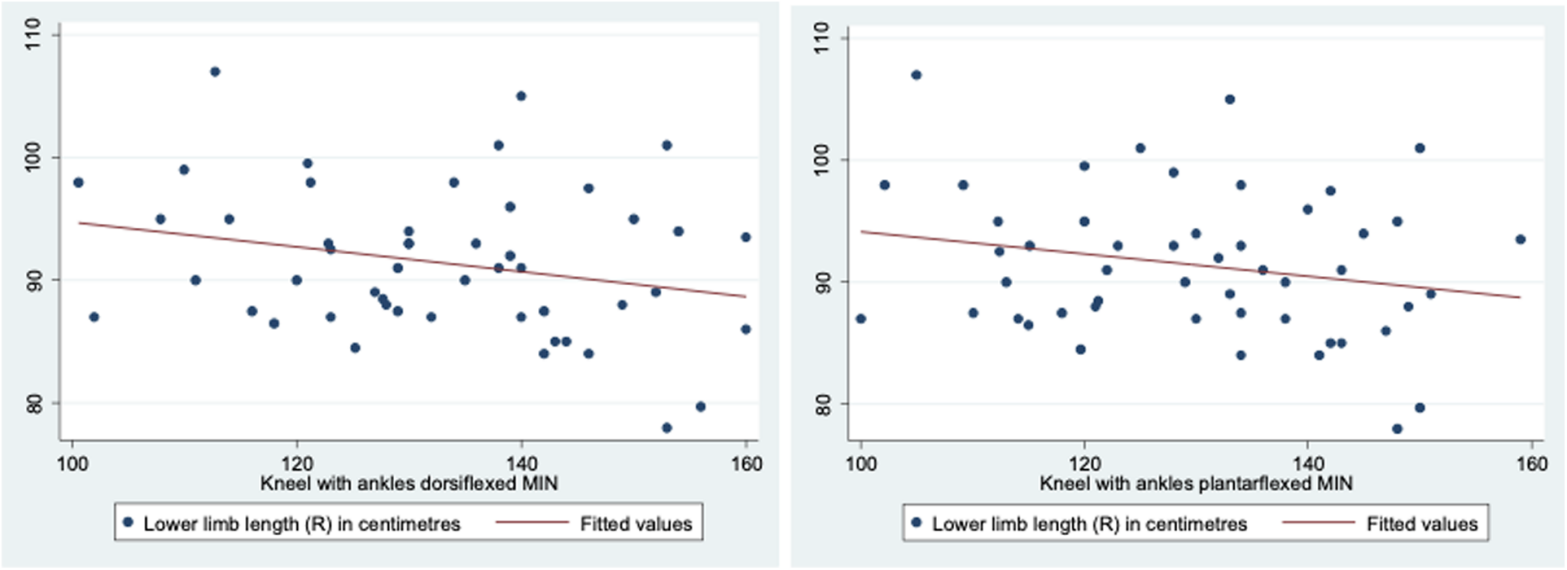
Correlation between lower limb length and knee range of movement during activities.

**Figure 7:**
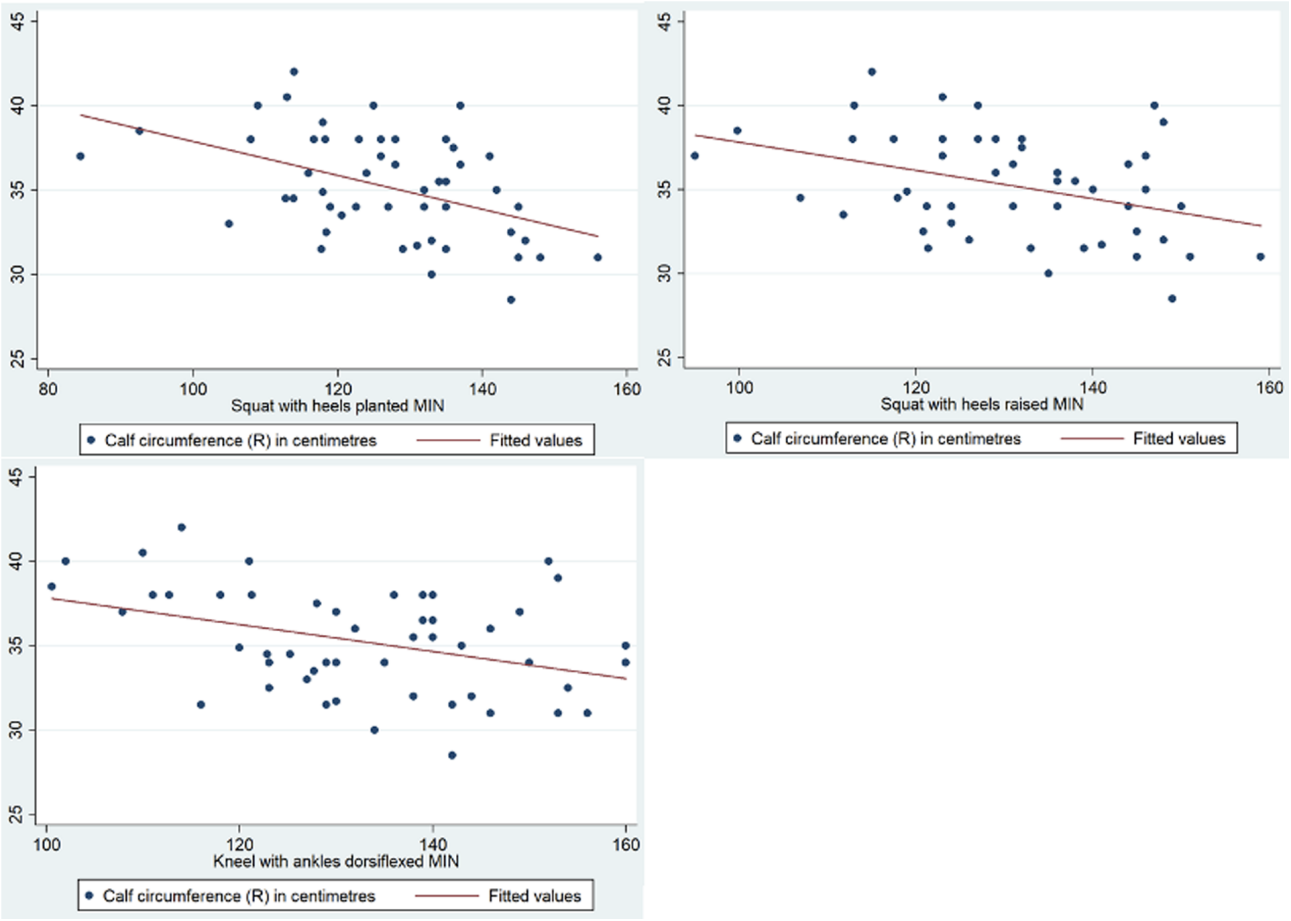
Correlation between calf circumference and knee range of movement during activities.

Further analysis was performed to evaluate the association between knee ROM during various activities and the anthropometric measurements of the participants. Leg length was a statistically significant factor affecting knee ROM when walking on flat ground [R(48) = −0.317, *p* = 0.0248], descending stairs (*p* < 0.001, 95% CI −5.47 to −2.15), squatting with heels planted (*p* = 0.001, 95% CI −3.79 to −1.13), squatting with heels raised (*p* = 0.001, 95% CI −3.73 to −0.954), kneeling with ankles dorsiflexed (*p* < 0.001, 95% CI −4.62 to −1.98), and kneeling with ankles plantarflexed (*p* < 0.001, 95% CI −4.57 to −1.47). A graphical representation of the correlation is shown in Figure 5. Lower limb length was also identified as a statistically significant factor affecting knee ROM when kneeling with ankles dorsiflexed (*p* = 0.035, 95% CI 0.0690–1.83) and ankles plantarflexed (*p* = 0.028, 95% CI −0.108 to 1.77). A graphical representation of the correlation is shown in Figure 6. Finally, calf circumference was another statistically significant factor affecting knee ROM when squatting with heels planted (*p* = 0.010, 95% CI −2.74 to −0.387), heels raised (*p* = 0.012, 95% CI −3.34 to −0.429), and kneeling with ankles dorsiflexed (*p* = 0.013, 95% CI −3.32 to −0.405). A graphical representation of the correlation is shown in Figure 7.

A summary of the knee ROM required for the various activities and the anthropometric measurements that influence the ROM is presented in Table 2.

**Table 2:** Movements and knee flexion.

Movements (°)	All, mean (SD)	Male, mean (SD)	Female, mean (SD)	Anthropometric factors influencing knee ROM
Walking on flat ground	55.7 (8.76)	55.5 (8.06)	56.0 (10.1)	Leg length: R(48) = −0.317, *p* = 0.0248
Ascend stairs	68.2 (12.0)	65.7 (8.82)	72.6 (15.5)	None
Descend stairs	70.9 (16.6)	70.7 (13.7)	71.2 (21.2)	Leg length: *p* < 0.001 (95% CI −5.47 to −2.15)
Squat with heels planted	127 (14.1)	124 (14.5)	130.8 (12.5)	Leg length: *p* = 0.001 (95% CI −3.79 to −1.13) Calf circumference: *p* = 0.010 (95% CI −2.74 to −0.387)
Squat with heels raised	131 (14.0)	130 (13.8)	133 (14.3)	Leg length: *p* = 0.001 (95% CI −3.73 to −0.954) Calf circumference: *p* = 0.012 (95% CI −3.34 to −0.429
Kneel with ankles dorsiflexed	133 (15.0)	132 (16.0)	135 (13.3)	Lower limb length: *p* = 0.035 (95% CI 0.0690–1.83) Leg length: *p* < 0.001 (95% CI −4.62 to −1.98) Calf circumference: *p* = 0.013 (95% CI −3.32 to −0.405)
Kneel with ankles plantarflexed	130 (14.4)	130 (14.3)	129.5 (15.1)	Lower limb length: *p* = 0.028 (95% CI 0.108–1.77) Leg length: *p* < 0.001 (95% CI −4.57 to −1.47)
Sit cross-legged	106 (12.6)	106 (11.6)	105.7 (14.5)	None

## Discussion

The findings from this study provide important insights into the knee ROM required to perform daily activities comfortably in Singapore, an urban, multicultural, Southeast Asian city. The data, based on a population with urban and cultural demands, suggest that activities such as squatting, kneeling, and sitting cross-legged, which are more prevalent in Southeast Asia, demand a high degree of knee flexion. This study addresses a gap in the literature, as limited data exist regarding the specific ROM requirements of non-Western populations, particularly in Southeast Asia, where unique cultural practices impose different demands on TKA biomechanics.

### Patient expectations after total knee arthroplasty

One key implication of this study relates to the expectations of patients undergoing TKA. Prior research has shown that insufficient post-operative knee flexion significantly contributes to patient dissatisfaction after TKA (8, 13). In Singapore's urban environment, stairs are a common feature of public spaces, including public transport (e.g., boarding and alighting buses), and residential areas, where more than 70% of Singaporeans live in high-rise public housing—some of which lack direct lift landing. In our study, we found that the minimum knee ROM values required for ascending and descending stairs were 68.2°–70.9°, respectively. Previous studies reported that ascending stairs required knee flexion ranging from 92.9° to 104.1°, while descending stairs required 86.9°–104.1° (14–16). The step height reported in those studies ranged from 16.5 to 18 cm, while the step height in our study was 15 cm, likely contributing to the lower knee flexion requirement observed in this study. Considering that typical post-operative ROM after standard TKA procedures ranges from 95° to 123° (17, 18), patients can be counselled that they should be able to perform stair climbing after surgery. However, other factors, such as hip and ankle ROM and the height of an individual, need to be considered during pre-operative counselling.

Although sufficient for walking on flat ground and stair climbing, the ROM achieved after TKA may not be sufficient for kneeling and squatting. In our study, kneeling with ankles dorsiflexed required a knee flexion of 133°–and kneeling with ankles plantarflexed required a knee flexion of 130°. Previous studies have reported values ranging from 141° to 153° (19–21). The discrepancy could be due to differences in kneeling positions. For squatting, our study found that squatting with heels planted required a minimum knee flexion of 127°–and squatting with heels raised required a minimum knee flexion of 131°. Our results are comparable to those from other studies reporting values of 125° (19, 22). Given the difference between post-operative TKA ROM (17, 18) and the ROM observed for squatting and kneeling in the current study and others (19–22), the expectations in returning to such cultural practices need to be moderated.

As squatting and kneeling are more commonly performed in the cultural context of Southeast Asia, these findings underscore the need for surgeons to consider cultural and functional expectations and counsel patients accordingly. This approach can help better align patient expectations and improve satisfaction following TKA.

### Anthropometric factors affecting knee range of motion

Another important finding is the correlation between specific anthropometric measurements and knee ROM. Leg length, lower limb length, and calf circumference were significantly associated with the knee ROM required for various movements. Notably, an inverse relationship was observed between leg length and the minimum knee flexion required for activities, a finding previously reported in the literature (23). Individuals with longer limbs require less flexion to clear a step. This insight is particularly relevant for Southeast Asian patients, who, on average, are shorter than Caucasian patients (24) and may therefore require a greater knee ROM to navigate daily activities. The need for greater knee flexion has even led to the development of high-flexion TKA implants to cater to patients (25, 26). Similarly, an inverse relationship was observed between calf circumference and knee ROM during squatting and kneeling. It is likely that the knee ROM was limited by thigh-calf contact (27). Of note, there was no significant correlation between calf circumference and ROM during walking and stair climbing, which was likely due to the smaller ROM required for these activities.

Overall, the results suggest that body dimensions play a role in determining the degree of knee flexion achievable during various activities. Clinicians should consider these measurements into account when evaluating functional needs of patients and planning for surgery, particularly when discussing the anticipated recovery of knee flexion post-TKA. This is especially relevant for Southeast Asian patients who may have different body morphologies compared to Caucasian populations, where much of the existing literature on knee ROM has been derived. However, the authors would like to highlight that the biomechanics of the knee and lower limb is complex and dedicated studies involving more advanced measurement systems are required to accurately determine the relationships between body dimensions and knee joint movement.

## Limitations

While this study provides new insights, it has several limitations. The sample size in this study was relatively small and may not be representative of the broader Southeast Asian region. Participants with undeclared pre-existing pathology might have been included and therefore affected the range of motion observed. In addition, the range of movement was recorded with a simple device compared to other studies that used more complex systems.

In conclusion, the ROM after TKA is more likely to meet the demands of urban city living, such as stair climbing, than cultural practices like squatting and kneeling. The findings of this study, combined with an understanding of cultural expectations, might prove to be valuable for counselling and managing patients undergoing TKA in Singapore and other countries in the region with a similar urban landscape.

## Data Availability

The raw data supporting the conclusions of this article will be made available by the authors without undue reservation.
